# A 30-Year Journey Through Integrative Physiology Research and Education, Courtesy of Angiotensin II

**DOI:** 10.3389/fphys.2021.785878

**Published:** 2021-12-13

**Authors:** Kathleen S. Curtis

**Affiliations:** Center for Health Sciences, Oklahoma State University, Tulsa, OK, United States

**Keywords:** angiotensin II, estrogen, body fluid balance, graduate training, medical education

## Summary

This brief article is a personal perspective on the importance of integrative physiology education and research, using angiotensin II as an example of how integrative physiology occurs and why it matters. It also offers suggestions and strategies to ensure that integrative physiology remains vibrant and appreciated.

Picture it: University of Pittsburgh, circa 1994. Graduate students in a lab are discussing findings from recent experiments, leading to the idea that maintaining body fluid balance requires second-by-second monitoring of the post-ingestive consequences of water intake on body fluid status, and the real-time adjustment of ongoing intake in the face of those consequences. It was a behavioral neuroscience lab, after all, and the research questions fell under the umbrella of how the brain coordinated physiological and behavioral processes to ensure survival in the face of internal and external challenges. A student from a nearby lab that employed patch-clamp recording to investigate ionic conductances and their role in long-term potentiation in hippocampal neurons entered the lab. The spirited discussion continued until, recognizing the befuddled look on the face of the newcomer, the senior graduate student said “I’m sorry. We get carried away with this big-picture stuff.” The newcomer laughed a bit uncertainly and replied “Hey, to me the **cell** is the big picture.”

This was nearly 30 years ago, when the technological revolutions that allow researchers to investigate the molecular bases of cellular function and drill down further and further into the regulation of the genome itself were in their infancy. Even then, however, there was the perception that those who studied integrative physiology were the red-headed stepchildren of the scientific world, while those who used complex techniques to investigate (intra)cellular processes attained almost mythical status, striding the globe like colossuses. I’m sure it is obvious that I was in the former group. What may be less obvious is that attitudes haven’t changed that much in the intervening years. To be clear, I am absolutely not arguing that cellular/molecular/genetic approaches to investigate mechanisms underlying physiological function are not of value or that they have not advanced our knowledge of physiology. But I ***would*** argue that both research and education have moved so far in the direction of cellular/molecular/genetic biology that comprehension of basic physiology, in general, and integrative physiology, in particular, have suffered. This is bad enough for physiology research, but medical education also has shifted to a more cellular/molecular/genetic focus which can readily be seen by leafing through physiology textbooks. Indeed, many United States medical schools no longer require a physiology course for admission, but consider it an elective, along with cell and molecular biology [see Association of American Medical Colleges (AAMC), 2021]. The long-term impact that derives from inadequate understanding of integrative physiological functioning among healthcare providers remains to be seen.

Having been trained in behavioral neuroscience by advisors with an appreciation for and encyclopedic knowledge of the physiological underpinnings of body fluid balance, integrative physiology was an integral part of my graduate training and research. I still clearly recall the specific paper that put it into perspective for me: Robinson and Evered, 1987. These investigators held blood pressure constant to assess the effects of angiotensin manipulations on water intake, recognizing that manipulations of the renin-angiotensin system frequently employed in studies of drinking behavior also affect variables such as blood pressure and renal sodium handling. Such unintended consequences could, in turn, alter drinking, thereby dictating the need for more cautious interpretation of the data—and for more thoughtful experimental design (see also, Evered, 1992). It was a “light bulb moment” for me. “Of course!” I thought. “We’re investigating a system that, by definition, integrates interrelated complementary neural, cardiovascular, renal, and endocrine elements to elicit physiological and behavioral responses intended to compensate for body fluid challenges.” In fact, I attempted to map it all out, an effort that required more sophisticated artistic skills than I possessed at the time. In retrospect, my response seems overly simplistic; it was (or should have been) patently obvious, rather than the light bulb moment I perceived it to be. Nonetheless, it was a perspective that informed my graduate research and that I strive to incorporate into my own research about the central effects of estrogen on body fluid balance.

But as time has passed, I find that students come into the classroom and into the lab with an impressive amount of knowledge about the molecular biology of angiotensin signaling but far less knowledge of the outcome of that signaling. Admittedly, the sophisticated techniques now available allow us to dig ever deeper into intracellular pathways such as those involved in angiotensin signaling. Indeed, there are numerous comprehensive reviews of angiotensin receptor activation (e.g., Forrester et al., 2018) that focus on intracellular signaling molecules associated with angiotensin receptors in physiological systems ranging from renal to immune. However, the majority of these papers appear to rely on the reader to fill in the blanks about the significance of this signaling. For those of us trained as integrative physiologists, filling in the blanks of the multi-system effects of angiotensin II may be easier. We understand the neural, cardiovascular, renal, and endocrine impacts of angiotensin receptor activation. But for students and trainees who haven’t had the benefit of this perspective in their education or in their research training, the blanks may remain in this reduced perspective. As a result, their big picture is, quite simply, smaller.

This is not to say that a reductionist approach is intrinsically wrong or bad. Complex systems must be reduced if research is to be tractable. However, when the point of view is reduced along with the approach, we may be amassing more and more information about the cellular, molecular, and genetic mechanisms that underlie individual physiological functions, while losing our understanding of the role of each function, let alone how the functions are integrated to promote the survival of the whole animal. In other words, absent the perspective provided by integrative physiology, the bigger picture trends increasingly toward being the cell.

**FIGURE 1:**
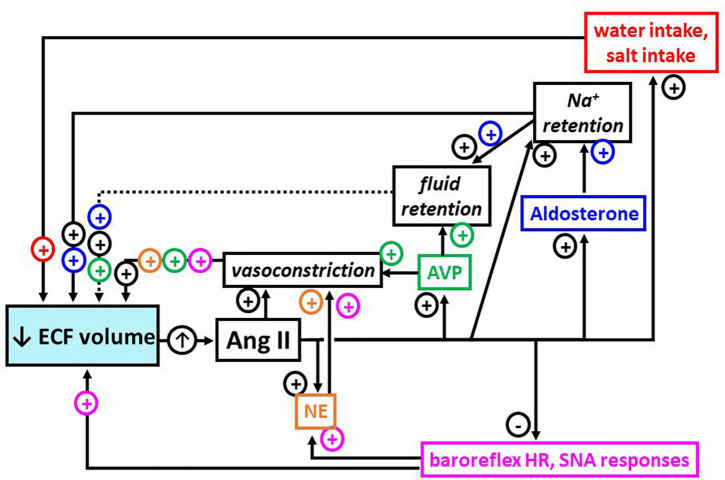
Multi-system, integrated responses after release of angiotensin II (Ang II; up arrow) stimulated by loss of extracellular fluid (ECF) volume (blue box, down arrow). Arrows indicate effects stimulated (+) or inhibited (−) by Ang II and their positive impact on fluid volume, directly or *via* increased blood pressure; dashed arrow indicates minor role in ECF volume control. Together, these responses are integrated to maintain blood flow to organs and tissues in the face of ECF volume loss. As indicated by black font, boxes, and + signs, Ang II has direct actions that contribute to restoring ECF volume. In addition, as indicated by pink, gold, green, blue, and red font, boxes and + or − signs, Ang II has actions on a number of other effectors that indirectly serve to restore ECF volume (pink = baroreflex heart rate (HR), sympathetic nerve activity (SNA) responses; gold = norepinephrine (NE); green = arginine vasopressin (AVP); blue = aldosterone; red = water intake, salt intake).

How do we address this? As educators we can emphasize integrative physiology and promote integrative thinking in our entry level and advanced physiology courses (see, e.g., Curtis et al., 2020). In addition, we can write or contribute to textbooks to do the same, while incorporating the concept of integrated functioning into our undergraduate, graduate, and medical curricula as a whole. As researchers, we must educate ourselves and our trainees about integrative physiology and about how interactions among physiological systems can influence our findings. There are undoubtedly papers that provided light bulb moments for all of us, papers that profoundly influenced not only our research, but also our thinking about the field in which we work. I make sure to discuss the Robinson and Evered paper with trainees, particularly new trainees and most especially with undergraduate students. But we also must keep in mind interactions among physiological systems while interpreting our data and ensure that our students do too, so that we do not overinterpret or overgeneralize from those findings. To help my students and trainees appreciate the importance of this, I also provide a diagram I devised (Figure 1) illustrating the complex interrelatedness of the effects of angiotensin II. I should note, however, that while drawing software made it possible for me to generate this diagram, I still don’t possess the skills to create an illustration summarizing *all* the changes that occur after loss of extracellular fluid volume; thus, the focus remains on angiotensin.

Grasping the concept of integrated, complementary responses that underpin the role of angiotensin II in body fluid regulation is critical for my laboratory’s studies of estrogen effects on body fluid regulation. These studies must take into account that estrogen has both peripheral and central actions that may influence any of the angiotensin effects shown in Figure 1. As only a few examples, estrogen actions may be exerted by directly affecting end organs (e.g., Suzuki and Kondo, 2012) or by altering biosynthesis and/or release of the hormones of body fluid regulation (e.g., Hartley et al., 2004). Moreover, estrogen may alter angiotensin synthesis (e.g., Gallagher et al., 1999) and/or sensitivity by up- or down-regulating receptors for angiotensin II (e.g., Kisley et al., 1999; Krause et al., 2006).

I use my own research as an example of the need for students to have an appreciation of integrative physiological systems in order to design and interpret their studies—and the complexity of our studies further illustrates the necessity for integrated physiology research and education. Perhaps more to the point, however, increased understanding of this aspect of integrative physiology will inform other areas, building upon knowledge gained to better understand the bigger picture in the lab. Ultimately, the goal is to incorporate that knowledge into the classroom so that, in the end, it can be translated into improved health and healthcare.

## Data Availability Statement

The original contributions presented in the study are included in the article/supplementary material, further inquiries can be directed to the corresponding author/s.

## Author Contributions

KC wrote the manuscript, developed the figure, and approved the manuscript submission.

## Conflict of Interest

The author declares that the research was conducted in the absence of any commercial or financial relationships that could be construed as a potential conflict of interest.

## Publisher’s Note

All claims expressed in this article are solely those of the authors and do not necessarily represent those of their affiliated organizations, or those of the publisher, the editors and the reviewers. Any product that may be evaluated in this article, or claim that may be made by its manufacturer, is not guaranteed or endorsed by the publisher.
